# Thyroid hormone receptor orthologues from invertebrate species with emphasis on *Schistosoma mansoni*

**DOI:** 10.1186/1471-2148-7-150

**Published:** 2007-08-29

**Authors:** Wenjie Wu, Edward G Niles, Philip T LoVerde

**Affiliations:** 1 Department of Microbiology and Immunology, School of Medicine and Biomedical Science, State University of New York, Buffalo, NY 14214, USA; 2 Southwest Foundation for Biomedical Research, 7620 NW Loop 410 San Antonio, Texas, 78227-5301, USA; 3 Departments of Biochemistry and Pathology, University of Texas Health Sciences Center, San Antonio, Texas, 78229-3800, USA

## Abstract

**Background:**

Thyroid hormone receptors (TRs) function as molecular switches in response to thyroid hormone to regulate gene transcription. TRs were previously believed to be present only in chordates.

**Results:**

We isolated two TR genes from the *Schistosoma mansoni *and identified TR orthologues from other invertebrates: the platyhelminths, *S. japonium *and *Schmidtea mediterranea*, the mollusc, *Lottia gigantean *and the arthropod *Daphnia pulex*. Phylogenetic analysis of the DNA binding domain and/or ligand binding domain shows that invertebrate and vertebrate TRs cluster together, TRs from the vertebrates and from the jawless vertebrate (lamprey) clustered within separate subgroups, Platyhelminth TRs cluster outside of the vertebrate TR subgroups and that the schistosome TRs and *S. mediterranea *TRs clustered within separate subgroups.

Alignment of the C-terminus of the A/B domain revealed a conserved TR-specific motif, termed TR 'N-terminus signature sequence', with a consensus sequence of (G/P)YIPSY(M/L)XXXGPE(D/E)X.

Heterodimer formation between *S. mansoni *TRs and SmRXR1 suggests that the invertebrate TR protein gained the ability to form a heterodimer with RXR. ESMA analysis showed that SmTRα could bind to a conserved DNA core motif as a monomer or homodimer.

**Conclusion:**

Vertebrate TR genes originated from a common ancestor of the Bilateria. TR genes underwent duplication independently in the Protostomia and Deuterostomia. The duplication of TRs in deuterostomes occurred after the split of jawless and jawed vertebrates. In protostomes, TR genes underwent duplication in Platyhelminths, occurring independently in trematode and turbellarian lineages. Using *S. mansoni *TRs as an example, invertebrate TRs exhibited the ability to form a dimer with RXR prior to the emergence of the vertebrate TRs and were able to bind to vertebrate TR core DNA elements as a monomer or homodimer.

## Background

Thyroid hormones (TH) play important roles in growth, development and metabolism in vertebrates. TH is synthesized in the thyroid gland under the control of thyroid-stimulating hormone (TSH) secreted by the pituitary. TSH secretion is controlled by thyrotropin-releasing hormone (TRH) which is secreted from the hypothalamus. THs are lipophilic molecules able to passively cross the membrane and bind to its receptor, the thyroid hormone receptor (TR). TRs belong to a superfamily of transcription factors called nuclear receptor (NR) superfamily, based on protein sequence similarities, structural motifs and functionality [1]. TRs function as a molecular switch in response to the thyroid hormones T3 or T4 to activate or repress gene transcription depending on the promoter context and thyroid hormone binding status [2]. The typical nuclear receptor contains an N-terminal A/B domain, a conserved C domain (DNA binding domain, DBD), a D domain (hinge region) and a moderately conserved E domain (ligand binding domain, LBD). The most conserved DBD contains two zinc finger motifs (CI and CII). Like all NRs, TRs regulate transcription through its binding to the promoter region of a target gene by the DBD and they activate or repress mRNA synthesis through co-regulators bound to the LBD [1]. The specific target DNA sequence to which NRs bind is called a hormone response element (HRE). The typical HRE is a direct, inverted or everted repeat or palindrome of the DNA sequence AGGTCA. TRs can bind to the HRE as a monomer, a homodimer or as a heterodimer with RXR, another member of nuclear receptor superfamily which contributes to the specificity of the TR. TH binds to the LBD of TR which results in a conformational change in the C-terminus of the receptor. Corepressors then dissociated from the TR allowing coactivators to bind to the C-terminus of the TR in a hormone-dependent manner. TR and the coactivator complex activate the expression of the target gene [3,4].

TR was previously believed to be an innovation of chordates as the genomes of insects (Drosophila and mosquito) and nematodes (*Caenorhabditis elegans *and *C. briggus*) do not contain TR genes [5-8]. Recently, we identified two thyroid receptor homologues in the flatworm *Schistosoma mansoni *[9], one of which was found in the *S. mansoni *EST database [6,10]. The presence of TR homologues in *S. mansoni *demonstrated that the TR orthologue genes are present outside of chordates. However, it is still unclear whether these prostostome TRs possess the same functional domains as in vertebrate TRs. Another question is whether the TR orthologue is present in other invertebrates or just in the platyhelminth lineage? Answers to these questions will help to understand the origin of TR genes and evolution of the function of vertebrate thyroid hormone network. To begin to address these questions, we isolated cDNAs of *S. mansoni TRs *and demonstrated that TRs in platyhelminths are highly conserved not only in sequence similarity, but also in gene organization, protein-protein interaction and in DNA-binding ability. Furthermore, we mined the available genome data and demonstrated that TR orthologues are present in different invertebrate animals but not in Porifera or Cnidaria. Phylogenetic analysis showed that the TR orthologue likely originated from a common ancestor of the Bilateria.

## Results

### TR orthologue genes in invertebrate animals

**Figure 1:**
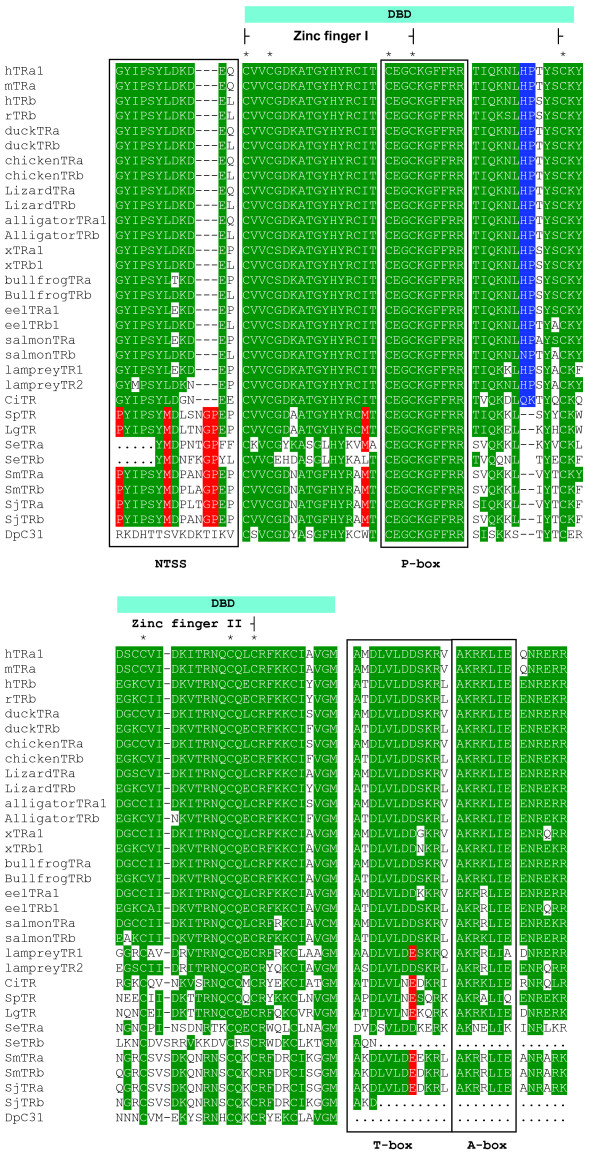
**Sequence alignment of the DNA binding domain (DBD) of TRs**. There is a highly conserved motif found at the 3' end of A/B domain, termed the 'N-terminal signature sequence' (NTSS). In non-chordate TR homologues, it is PYIPSYMXXXGPEEP; while in chordate NTSS, three amino acids are deleted (represented by dashes) generating a sequence of GYIPYL(D/E)KE(P/Q/L). In the N-terminus of the DBD, a methionine at position 16 (highlighted in red) is non-chordate TR specific. In this position an isoleucine is conserved for all chordate TRs. Two amino acids (His and Pro for all species except *Ciona*) are inserted at positions 33–34 of the N-terminus of DBD in chordate TRs (highlighted in blue). An aspartic acid in vertebrate TRs at the 8th position of the T-box is highly conserved for vertebrate TRs, a glutamic acid at this position is conserved for other TRs (highlighted in red). Stars identify the conserved cysteine residues that comprise the zinc finger of the DBD. The conserved residues are highlighted in green. CiTR: *Ciona intestinalis *nuclear receptor 1, DpTR: water flea *Daphnia pulex *TR, hTRa: Human thyroid receptor alpha, hTRb: Human thyroid receptor beta, LgTR: owl limpet *Lottia gigantean *TR, SeTRa: turbellarian *Schmidtea mediterranea *TRα, SeTRb: *S. mediterranea *TRβ, SjTRα: blood fluke *Schistosoma japonium *TR alpha, SjTRβ: *S. japonium *TR beta, SmTRα: blood fluke *S. mansoni *TR alpha, SmTRβ: *S. mansoni *TR beta, SpTR: sea urchin *Strongylocentrotu purpuratus *TR. The accession numbers of the aligned human nuclear receptors can be found in Additional files 1 and 2.

By an extensive search of available databases, predicted genes encoding TR orthologues were found in different invertebrate animals using the conserved DBD as a query. They include two genes in each of the platyhelminth species evaluated, the turbellarian *Schmidtea mediterranea *and the trematodes, *Schistosoma mansoni *and *S. japonium*, one each from the mollusc *Lottia gigantean *(owl limpet) and the crustacean *Daphnia pulex *(water flea) (Fig. 1 and see additional file 1). No TR orthologues were identified in any species of the Radiata.

### Sequence alignment analysis

Alignment of DBD amino acid sequences show that the invertebrate TR orthologues are highly conserved (Fig. 1). All TRs (from chordate and non-chordate species) possess an identical P-box sequence, which determines the specificity of target DNA binding. The methionine at position 16 of the N-terminus of the DBD (highlighted in red in Fig. 1) was non-chordate TR-specific. Instead at this position an isoleucine is conserved in all chordate TRs examined to date. Two amino acids (His and Pro) at positions 33–34 of N-terminal of DBD were chordate TR-specific (highlighted blue in Fig. 1). The second zinc finger is less conserved in non-chordate TR orthologues compared to the first zinc finger, but still shows a high degree of conservation (Fig. 1). The T-box and A-box which are located in the C-terminal extension (CTE) of the DBD are also highly conserved in non-chordate TR orthologues. They show 75–83% and 57–100% identity to the chordate T-box and A-box, respectively. These regions in the water flea TR orthologue are highly divergent, probably due to a recombination event that happened in this region (see below). An aspartic acid in the vertebrate TRs at the 8th position of N-terminus of the T-box is highly conserved for jawed vertebrate TRs, a glutamic acid at this position is conserved in other TRs. Although the N-terminal A/B domain of nuclear receptors is divergent, there is a highly conserved motif found at the 3' end of the TR A/B domain, we termed the 'N-terminal signature sequence' (NTSS). The TR NTSS is a consensus sequence of (G/P)YIPSY(M/L)XXXGPE(D/E) (Fig. 1). In non-chordate TR orthologues, the sequence is PYIPSYMXXXGPEEP; while in chordates, three amino acids are deleted generating a sequence of GYIPYL(D/E)KE(P/Q/L). The deleted three amino acids in chordate NTSS were deduced from the non-chordate sea urchin TR (SpTR). Since the deuterostome SpTR shares the same NTSS with that of protostome TRs, this suggests that the non-chordate NTSS is the primitive state.

### Functional domains of non-chordate TRs

To determine the functional domains of a non-chordate TR orthologue, cDNAs encoding the entire open reading frame (ORF) of the two *S. mansoni TRs *(*SmTRα *and *SmTRβ*) were isolated, sequenced and the sequences deposited in GenBank (*SmTRα *[GenBank:AY395038, AY395059–AY395063, AH013464) and *SmTRβ *[GenBank:AY395039]).

**Figure 2:**
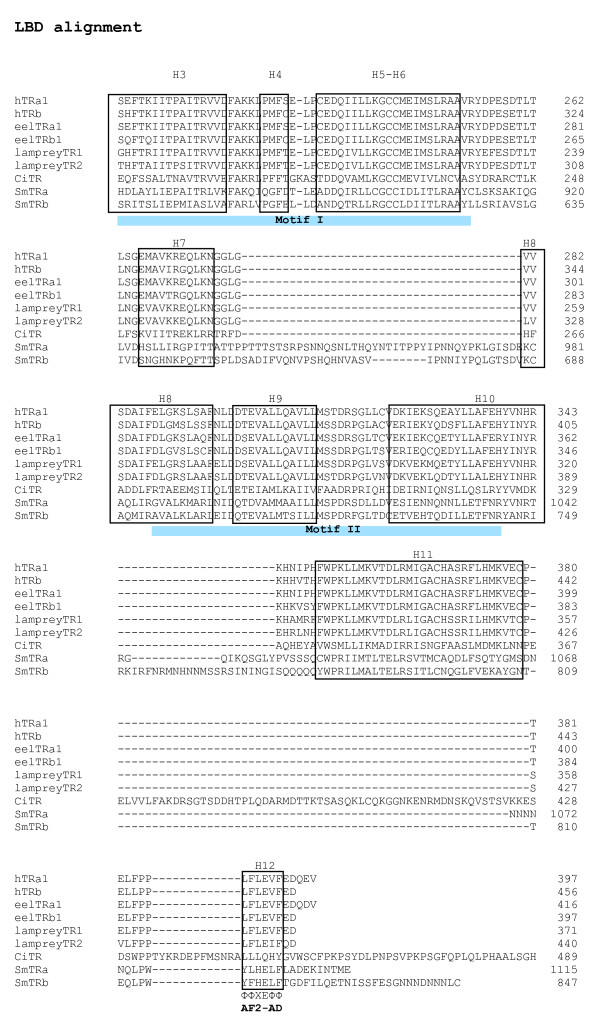
**Sequence alignment of the ligand binding domain (LBD) of *S. mansoni *TRα and SmTRβ**. Alignment of sequences from Helices (H) 3–12 of the LBD domain of *S. mansoni *TRs (SmTRα and SmTRβ). Helices described in [53] are boxed. The autonomous activation domain (AF2-AD) is indicated. Numbers at the end of each line indicate residue positions in the original sequence, amino acids of CiTR 490–587 are not shown in the alignment. CiTR: *Ciona intestinalis *nuclear receptor 1, hTRa: Human thyroid receptor alpha, hTRb: Human thyroid receptor beta, SmTRa: blood fluke *S. mansoni *TR alpha, SmTRb: *S. mansoni *TR beta. The accession numbers of the aligned human nuclear receptors can be found in Additional file 2.

**Figure 3:**
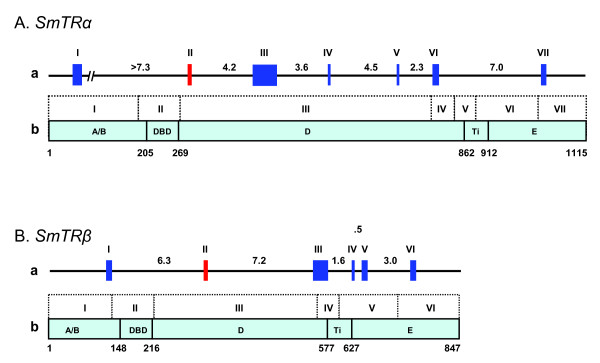
**Gene organization of *SmTRα *and *SmTRβ***. **A. *SmTRα*. B. *SmTRβ*. (a) **Showing exons and size of introns; Roman numerals indicate exons. **(b) **Showing the size of exons and their correspondence to the different protein domains. A/B: A/B domain, DBD: DNA binding domain, D: D domain (hinge region), Tτ: signature sequence of the LBD, E: E domain (LBD) after Tτ.

*SmTRα *and *SmTRβ *encode proteins of 1115 amino acids and 847 amino acids, respectively. Each protein exhibits a modular structure characteristic of the nuclear receptor superfamily with an A/B domain, a conserved DBD, a hinge region and a LBD (Fig. 2 and 3). Alignment of the sequences demonstrated that the LBD is less conserved. Helices 1–2 in the LBD are highly divergent, similar to that of other reported schistosome NRs [11-19]. Although the LBD is less conserved (Fig. 2), the consensus motif I (from helix 3 to helix 6) and the consensus motif II (from the middle of helix 8 to the middle of helix 10) are conserved [20] (Fig. 2). A putative AF2 activation domain core (AF2-AD) (helix 12) is present in both SmTRs (Fig. 2). It exhibits a high degree of conservation (represented by YLHELF and YFHELF in SmTRα and SmTRβ, respectively) in comparison to the common consensus AF2-AD core structure of ΦΦXEΦΦ, where Φ denotes a hydrophobic residue [12,21,22].

**Figure 4:**
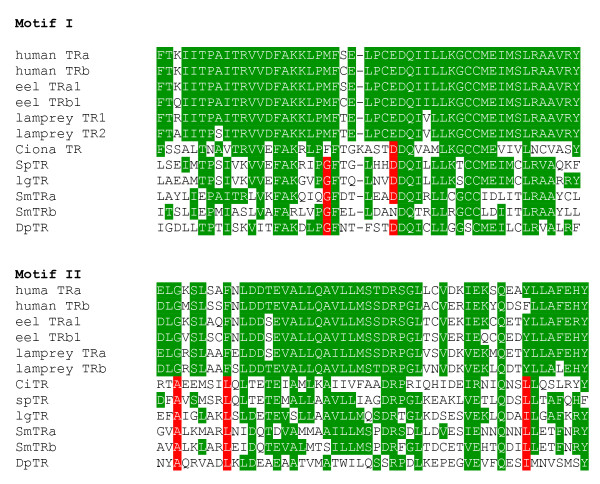
**Sequence alignment of motif I and motif II in the LBD**. Alignment of motif I and motif II in the LBD of TRs shows that two amino acids in motif I (a glycine at position 21 and an aspartic acid at position 29) and three amino acids in motif II (an alanine at position 3, a leucine at position 9 and an isoleucine or leucine at postion 45) are invertebrate TR homologue-specific (all highlighted in red). The conserved residues are highlighted in green. DpTR: water flea *Daphnia pulex *TR, LgTR: owl limpet *Lottia gigantean *TR, SmTRα: blood fluke *S. mansoni *TR alpha, SmTRβ: *S. mansoni *TR beta, SpTR: sea urchin *Strongylocentrotu purpuratus *TR. The accession numbers of the aligned human nuclear receptors can be found in Additional files 1 and 2.

The two consensus LBD motifs (motif I and motif II) were examined in other non-chordate TR orthologues when their DBD-containing contig contained these regions (Fig. 4). Two amino acids in motif I (a glycine at position 21 and an aspartic acid at position 29) and the three amino acids in motif II (alanine at position 3, a leucine at position 9 and an isoleucine or leucine at postion 45) were found to be invertebrate TR-specific (Fig. 4).

### Phylogenetic analysis

**Figure 5:**
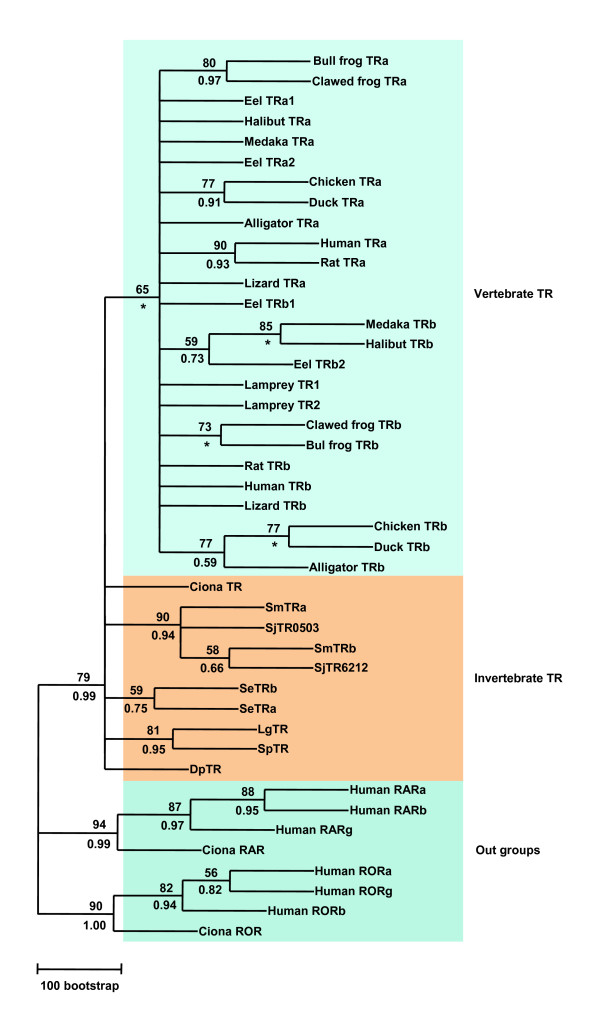
**Maximum Likelihood phylogenetic tree derived from amino sequences of the DNA binding domain**. A phylogenetic tree was constructed using the Maximum Likelihood (ML) method under Jones-Taylor-Thornton (JTT) substitution model with a gamma distribution of rates between sites (eight categories, parameter alpha, estimated by the program) using PHYML (v2.4.4)). Support values for the tree were obtained by bootstrapping a 100 replicates and are indicated above each branch. Branches under the bootstrap value of 50 were shown as polytomies. The same data set was also tested by Bayesian inference with a JTT amino acid replacement model + gamma rates. The trees were started randomly with four simultaneous Markov chains running for 5 million generations. Bayesian posterior probabilities (PPs) were calculated using a Markov chain Monte Carlo (MCMC) sampling approach implemented in MrBAYES v3.1.1, the PPs values are shown below each branch. Star indicates the node obtained by Bayesian inference which was different from that obtained by ML method. DpTR: water flea *Daphnia pulex *TR, LgTR: owl limpet *Lottia gigantean *TR, SeTRa: turbellarian *Schmidtea mediterranea *TRα, SeTRb: *S. mediterranea *TRβ, SjTRα: blood fluke *Schistosoma japonium *TR alpha, SjTRβ: *S. japonium *TR beta, SmTRα: blood fluke *S. mansoni *TR alpha, SmTRβ: *S. mansoni *TR beta, SpTR: sea urchin *Strongylocentrotu purpuratus *TR. The accession number of each sequence used for phylogenetic analysis can be found in Additional files 1 and 2.

Phylogenetic analysis of DBD sequences shows that all invertebrate TRs cluster with the vertebrate TR subgroup (Fig. 5), suggesting that they originated from a common ancestor gene. The two TR orthologues in the Platyhelminths, the turbellarian *S. mediterranea *and the trematodes (*S. mansoni *and *S. japonicum*) clustered outside of the vertebrate TR subgroups suggesting that they underwent duplication after the split of Prostostomia and Deuterostomia. Furthermore, each of the two duplicated trematode TR homologues cluster together within a subgroup, while the two turbellarian (*S. mediterranea*) TR homologues clustered within another subgroup. This result suggests that the TR duplication in the Platyhelminths occurred after the split of the trematodes and the turbellarians.

**Figure 6:**
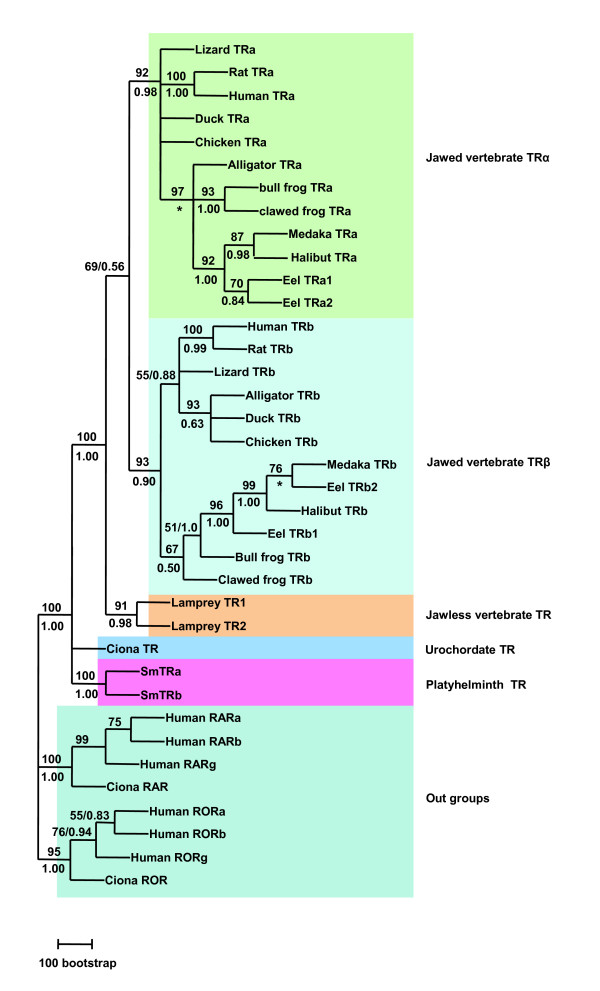
**Maximum Likelihood phylogenetic tree derived from amino sequences of DBD and LBD**. The phylogenetic relationship among TRs was examined by the Maximum Likelihood method (ML) and Bayesian inference with the same methods as in Fig. 6. Support values for ML tree were obtained by bootstrapping a 100 replicates and are indicated above each branch. Branches under the bootstrap value of 50 were shown as polytomies. Bayesian inference was running 3 million generations. The PPs are shown below each branch or after the ML bootstrapping value separated by a slash. Star indicates the node obtained form by Bayesian inference which was different from that obtained by the ML method. SmTRα: blood fluke *S. mansoni *TR alpha, SmTRβ: *S. mansoni *TR beta. The accession number of each sequence used for phylogenetic analysis can be found in Additional files 1 and 2.

A high resolution phylogenetic tree was constructed using the DBD and LBD sequences (Fig. 6). Since the only full length LBD sequence was identified from *S. mansoni *TRs, the *S. mansoni *TRs were used to represent the protostome TRs. The results show that all jawed vertebrate TRs clustered within a group including TRα and TRβ subgroups. The two jawless vertebrate lamprey TRs clustered outside of the jawed vertebrate TRs. This suggests that the vertebrate TR gene underwent duplication after the split of jawless and jawed vertebrates. The urochordate Ciona TR and the two duplicated trematode SmTRs cluster outside of the vertebrate TR groups. These results confirmed those obtained from the DBD analysis which suggested that the TR gene duplication occurred independently in vertebrates and invertebrates.

### Genomic organization

The gene organization of the TR homologue genes in *S. mansoni *(*SmTRα *and *SmTRβ*) was identified by alignment of the cDNA sequence with the genomic sequence (Fig. 3). The genomic sequences were obtained by screening *S. mansoni *bacterial artificial chromosome (BAC) libraries (SmBAC1 or CHOR103) and sequencing the BAC DNA [23], and by Blast searching WTSI *S. mansoni *WGS database [24]. Three clones (SmBAC1 3A6, 4G3 and 9D10) for *SmTRα *and 3 clones (CHOR103 3I15, 7K22 and 21F18) for *SmTRβ *were identified by screening the BAC libraries. A gDNA contig (Sm00.scaff00056.0030.1) for *SmTRα *and a gDNA contig (Sm00.scaff00631.0050) for *SmTRβ *were obtained from WTSI *S. mansoni *WGS database. The Contig Sm00.scaff00056.0030.1 contains a partial coding region for *SmTRα*, BAC DNAs (SmBAC1 3A6 and 4G3) were sequenced to generate a contig which contained full coverage of *SmTRα *cDNA sequence.

The ORF of *SmTRα *is encoded by seven exons spanning over 32 kb and the ORF of *SmTRβ *is encoded by six exons spanning over 21 kb (Fig. 3). All splice donor and acceptor sites for the two genes fit the GT-AG role. The A/B domain, hinge and E domains (LBD) are each encoded by 2–3 exons, respectively. The DBD of both proteins is encoded by one exon (Fig. 3). The 5' UTR and 3'UTR in both *SmTRα *and *SmTRβ *were not determined due to a short 3'UTR in *SmTRα *which seems not to be full length and due to the genomic contig not covering the 5'- and 3'UTR for *SmTRβ*.

**Figure 7:**
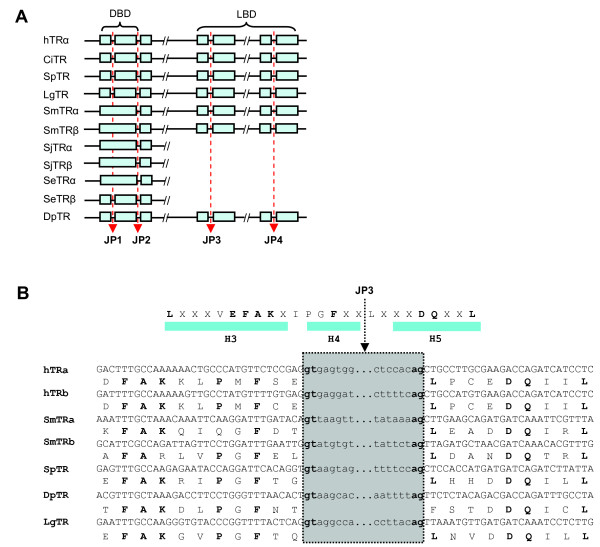
**Conserved junction sites within TRs. A**. Conserved junction site of TRs. JP1: Conserved junction position 1 which is within the DBD encoding region. JP2: Conserved junction position 2 which is at the end of the DBD coding region. JP3: Conserved junction position 3 which is within the LBD motif I coding region and is gene group specific. JP4: Conserved junction position 4 which is within the motif II of LBD encoding region. JP4 is conserved in all vertebrate NR genes. **B**. Shows the splice junction of invertebrate *TRs *within motif I which is at the same position as that found in all vertebrate TRs (NR1A) [20]. DBD: DNA binding domain, LBD: ligand binding domain. CiTR: Ciona NR1, DpTR: water flea *Daphnia pulex *TR, hTRα: Human thyroid receptor alpha, hTRβ: Human thyroid receptor beta, LgTR: owl limpet *Lottia gigantean *TR, SeTRα: turbellarian *Schmidtea mediterranea *TRα, SeTRβ: *S. mediterranea *TRβ, SjTRα: blood fluke *Schistosoma japonium *TR alpha, SjTRβ: *S. japonium *TR beta, SmTRα: blood fluke *S. mansoni *TR alpha, SmTRβ: *S. mansoni *TR beta, SpTR: sea urchin *Strongylocentrotu purpuratus *TR. H3: helix 3, H4: helix 4, H5: helix 5. The accession number of each sequence used for analysis can be found in Additional file 1.

It is known that there are four conserved splice junctions in nuclear receptor genes [3,4,9,20]. The first conserved junction position (JP1) is within the DBD encoding region, the second (JP2) is in the region which encodes the 3' end of the DBD, the third (JP3) is in the LBD motif I (also known as the LBD signature sequence, Tτ) encoding region [20] and the fourth one (JP4) is in the LBD motif II encoding region [20]. JP1 exhibits diversity among nuclear receptor genes but is conserved for certain gene groups including vertebrate TR genes [9]. JP2 is highly conserved usually in the region encoding the fourth amino acid of the hinge region, but most of the Drosophila NR genes have lost this junction [9]. JP3 is NR gene-specific [20] and JP4 is at the same position in all vertebrate NR genes [20]. All identified non-chordate TR orthologues possess the four JPs, except JP1 was lost in the schistsome TR genes (*SmTRα, SmTRβ, SjTRα *and *SjTRβ*) and JP2 was lost in *DpTR*. JP3 of all invertebrate TR genes is at the same position as that found in vertebrate TR genes (NR1A) (Fig. 7). JP4 in all invertebrate TRs is located at the same conserved position as found in all analyzed NRs [20]. The conserved splice junctions in these invertebrate TRs suggest that the organization of the TR genes was ancient and has been maintained throughout evolution. It adds further support to the notion that vertebrate and invertebrate TR orthologues originated from a common ancestor gene.

### Protein-protein interaction

**Figure 8:**
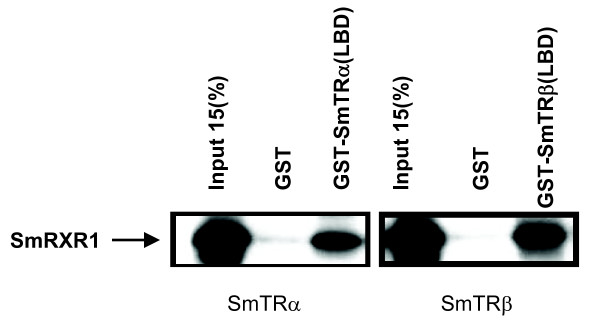
**Protein-protein interaction of SmTRs with SmRXR1**. GST pull down shows that both SmTRα and SmTRβ can form a heterodimer with SmRXR1 *in vitro*. ^35^S-labeled SmRXR1 was synthesized *in vitro *using pCITE-SmRXR1 as template and then incubated with GST-SmTRα(LBD), GST-SmTRβ(LBD) or GST (negative control) protein affixed to glutathione-Sepharose beads. The beads were collected, washed and the bound protein was resolved on 10% SDS acrylamide gel and visualized by autoradiography.

One property of vertebrate TRs is the ability to form a heterodimer with RXR [4]. To determine if a non-chordate TR would form a heterodimer with RXR, the interaction between SmRXR1 with SmTRα and SmTRβ was tested by GST pull-down experiments. Either GST-SmTRα-LBD or GST-SmTRβ-LBD fusion protein was incubated with *in vitro *translated ^35^S-SmRXR1. GST-bound resin was used as a negative control to assess non-specific background binding. The results showed that both SmTRα-LBD and SmTRβ-LBD could form a heterodimer with SmRXR1 *in vitro*. The results demonstrate that non-chordate TRs are capable of forming a heterodimer with RXR (Fig. 8).

**Figure 9:**
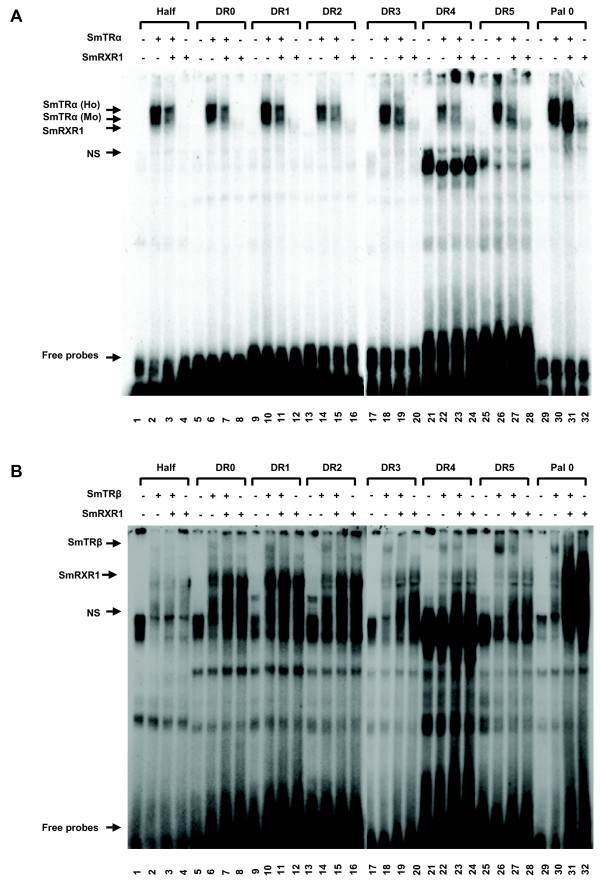
**DNA binding properties of SmTRs *in vitro***. Electrphoretic mobility shift assay (EMSAs) shows the DNA binding properties of SmTRs. A single protein or a combination of two proteins (SmTRα/SmRXR1 or SmTRβ/SmRXR1) were synthesized in a TNT quick coupled transcription/translation system (Promega) and allowed to bind to γ-^32^P labeled DNA elements containing a half-site, DR0-DR5 and Pal0. **A. SmTRα**. Lanes 1, 5, 9, 13, 17, 21, 25 and 29 contain lysate from the control transcription-translation reaction as negative controls. Lanes 2, 6, 10, 14, 18, 22, 26 and 30 contain lysate with *in vitro *translated SmTRα. Lanes 3, 7, 11, 15, 19, 23, 27 and 31 contain lysate with *in vitro *translated SmTRα and SmRXR1. Lanes 4, 8, 12, 16, 20, 24, 28 and 32 contain lysate with *in vitro *translated SmRXR1. NS: non-specific binding. **B. SmTRβ**. Lanes 1, 5, 9, 13, 17, 21, 25 and 29 contain lysate from the control transcription-translation reaction as negative controls. Lanes 2, 6, 10, 14, 18, 22, 26 and 30 contain lysate with *in vitro *translated SmTRβ. Lanes 3, 7, 11, 15, 19, 23, 27 and 31 contain lysate with *in vitro *translated SmTRβ and SmRXR1. Lanes 4, 8, 12, 16, 20, 24, 28 and 32 contain lysate with *in vitro *translated SmRXR1.. Ho: homodimer, Mo: monomer, NS: nonspecific binding.

Electrphoretic mobility shift assays (EMSAs) were performed to determine DNA binding specificity of SmTRα and SmTRβ. A DNA element containing the half-site AGGTCA, a direct repeat of the half-site spaced with 0–5 nucleic acids (DR0-DR5) and palindrome repeat of the half-site not separated by nucleic acids (Pal0) were employed. A gel shift was observed when γ-^32^P labelled half-site, DR0-DR5 or Pal0 were added to SmTRα. SmTRα could bind to all the tested oligonucleotides either as a monomer or as a homodimer (Fig. 9A). SmTRβ demonstrated a weak gel shift with the same labelled oligonucleotides as template (Fig. 9B). Heterodimers of either SmTRα or SmTRβ with SmRXR1 did not cause a gel shift (Fig. 9A and 9B). SmRXR1 alone could bind to the tested oligonucleotides (Fig. 9A and 9B) as we previously demonstrated [19].

**Figure 10:**
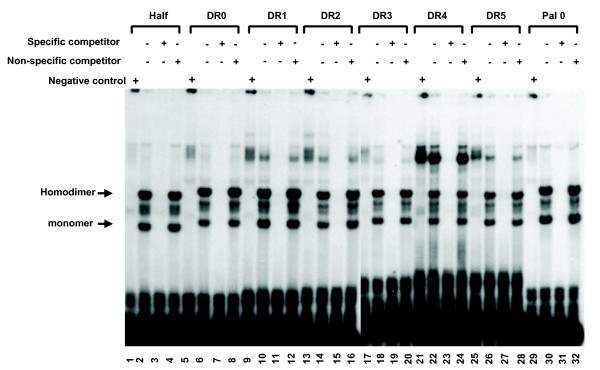
**DNA binding of SmTRα(Gln182-Ala288) *in vitro***. Electrphoretic mobility shift assay (EMSAs) shows the DNA binding properties of SmTRα(Gln182-Ala288). DNA binding of SmTRα(Gln182-Ala288) containing 20 aa at the 5' end of the DBD, the DBD and the 40 aa at 3' end of the DBD. Lanes 1, 5, 9, 13, 17, 21, 25 and 29: lysate from the control transcription-translation reaction as negative control. Lanes 2, 6, 10, 14, 18, 22, 26 and 30 contain with lysate with *in vitro *translated SmTRα (Gln182-Ala288). Lanes 3, 7, 11, 15, 19, 23, 27 and 31 contain lysate with *in vitro *translated SmTRα (Gln182-Ala288) with a 100 fold of cold specific competitor (unlabeled oligonucleiotides same as the labeled one). Lanes 4, 8, 12, 16, 20, 24, 28 and 32 contain lysate with *in vitro *translated SmTRα(Gln182-Ala288) with a 100 fold of cold non-specific competitor (5'-CGCGGATCCTGCAGCTCCAG-OH).

The above result showed that SmTRα could bind to a half site, DR0-DR5 and Pal0 element as a monomer or a homodimer. To test whether the DBD domain (which contains a weaker-dimer interface in the D-box) could bind to the oligonucleotides as a homodimer, *in vitro *synthesized SmTRα (Gln^182^-Ala^288^) (containing 20 aa at the 5' end of the DBD, the DBD and 40 aa at the 3' end of the DBD) was tested. SmTRα (Gln^182^-Ala^288^) bound to all tested oligonucleotides as either a monomer or a homodimer (Fig. 10). The results suggest that there is a dimer interface located in DBD region of SmTRα [25-27]. Specificity was demonstrated by the ability of cold specific competitor but not the non-specific competitor to prevent SmTRα binding to the oligonucleotide template.

## Discussion

An extensive search of whole genomic sequence (WGS) databases extracted from NCBI and online resources revealed the presence of TR orthologues in the Platyhelminths, Mollusca, Crustacea, and Echinodermata. However, no TR orthologues nor sequences encoding a TR P-box sequence of CEGCKG followed by the amino acid sequence FFRR (CEGCKGFFRR), which is unique to NR subfamily 1 including TR, was found in sponges (*Reniera sp*) nor cnidarians (*Hydra magnipapillata and Nematostella vectensis*). The results suggested that the TR orthologue gene originated in a common ancestor of the Bilateria. The gene duplication of *TRs *was deduced from a phylogenetic analysis employing DBD and LBD sequences. The analyses demonstrated that the TR orthologue genes underwent duplication independently in invertebrates and vertebrates. Furthermore, the TR orthologues in the Platyhelminths underwent duplication after the split of the turbellaria and trematodes. In vertebrates, the TR genes underwent a duplication event in lampreys, a jawless vertebrate, after the lamprey split from a common ancestor of jawed vertebrates. This result is consistent with the duplication of Hox gene clusters in lampreys [28]. Although the A/B domain of NR is highly divergent, we identified a conserved NTSS (N-terminal signature sequence, (G/P)YIPSY(M/L)XXXGPE(D/E)) located in the C-terminus of the A/B domain of TRs. Analysis of other NRs (data not shown) demonstrates this motif is found only in TRs suggesting it is TR-specific. The NTSS of TRs was found to be sequence-specific for the chordate and for the non-chordate TRs, three amino acids present in non-chordate TRs are missing in chordate NTSS. In the DBD, a methionine at position 16 of the N-terminus was non-chordate-specific, while two amino acids inserted at positions 33–34 of the N-terminus were chordate-specific, These differences which are conserved, support the notion that TR genes duplicated independently in non-chordate and chordate species.

That the JP3 conserved in all invertebrate TRs are also conserved in vertebrate TR genes support the notion that the vertebrate and invertebrate TR orthologue genes originated from a common ancestor gene. Further, that the four JPs are conserved in both invertebrate and vertebrate TR orthologue genes suggest that the gene organization of vertebrate TR genes is ancient and has been maintained through out evolution of TRs.

GST pull-down experiments showed that SmTRs exhibits similarity to vertebrate TR proteins as they both can form a heterodimer with RXR (SmRXR1). RXRs have been characterized in other invertebrates such as Mollusca and Arthropoda [29,30]. The formation of SmTRs with SmRXR1 indicates that other invertebrate TRs can possibly form a heterodimer with RXR. The ability of vertebrate NR to bind to a DNA element on a target gene was described as the 1–2–3–4–5 rule [31,32]. Vertebrate TRs are known to bind to half-sites as a monomer, to DR0 as a heterodimer with RXR [33] and to DR4 as a homodimer or heterodimer [32,34-43]. The binding of the TR monomer or homodimer is weak, which is due to a rapid dissociation of the TR-TRE complex [44]. Our EMSA show that SmTRα can bind to a half-site as a monomer and bind to DR0-DR5 as a homodimer. The results suggested that TR gained the ability to bind to conserved half-site repeats in a common ancestor, but did not subsequently evolve a distinct specificity as regards spacing between half sites. Although the DBD sequence between SmTRα and SmTRβ are highly similar, SmTRβ showed weak binding to the DNA elements compared to that of SmTRα. This suggests that other domains may have a role in determining the ability of SmTRβ to bind to the DNA element, such as has been shown for another schistosome nuclear receptor, SmNR1 [19]. Although both SmTRs could form a heterodimer with SmRXR1 as determined in a GST pull-down assay, SmTRα/SmRXR1 or SmTRβ/SmRXR1 did not bind to the DNA elements tested. This may due to the flanking or spacing of nucleotides between the HRE, as it is known that these nucleotides also determine the ability of the protein to bind to it. Identification of the HRE in a target gene will help to understand the evolution of TR DNA binding properties. Mammalian TRs are ligand-dependent transcription factors, they repress basal transcription through association with a variety of corepressors in the absence of T3. A structural change occurs when T3 binds to the TR that results in the release of natural corepressors and results in the recruitment of coactivators to activate gene transcription [4]. Determination of whether invertebrate TR can bind to T3 will help to understand the evolution of the thyroid hormone pathway.

## Conclusion

We have identified ortholouges of TR in various invertebrate phyla. Sequence analysis demonstrates that there is a 'N-terminal signature sequence' (NTSS) located in the A/B domain -(N-terminal region) specific to TRs. Phylogenetic analysis of conserved DBD sequences showed that TR orthologues originated from a common ancestor of the Bilateria which underwent gene duplication independently in the Protostomia and Deuterostomia. Using the schistosome TR orthologues as representative of the invertebrate TR genes, we have demonstrated that they can form heterodimers with RXR and bind as monomers and homodimers to consensus vertebrate DNA half sites and the direct repeat of the half sites DR0 to DR5.

## Methods

### cDNAs isolation

cDNAs containing the entire open reading frame (ORF) of two *S. mansoni TRs *(*SmTRα *and *SmTRβ*) were isolated by a PCR strategy using a *S. mansoni *paried adult worm cDNA library (pBluescript SK (+/-) phagemid) pool as template DNA. The PCR primers for one end (either 5' or 3' end) were designed according to a fragment encoding the previously identified DBD region of each gene [9]. The primer for the other end (either 5' or 3' end) was a vector universal primer (M13-Rev and T3, or M13-For and T7 primers). PCR products were separated on 1.2% agarose gels, ligated into pCR2.1 TOPO vector (Invitrogen) and sequenced. After the correct fragments were identified, the cDNA sequence containing the 5' UTR, ORF and 3' UTR were obtained by PCR. Each cDNA was shown to be related to a single mRNA species by sequencing the PCR products obtained from single-stranded cDNA using primers located within the 5'UTR and 3'UTR of each gene.

### Database search

Whole genomic sequences (WGS) were extracted from the GenBank public ftp site [45] and imported into the StarBlast program (DNASTAR) to build a local database [18] which was searched by tblastn using the sequence of the DBD of SmTRα as the query. Any sequence that contained a zinc finger structure of the DBD (Cys-X2-Cys-X13-Cys-X2-Cys or Cys-X5-Cys-X9-Cys-X2-Cys) was retained. Sequence walking was carried out to assemble the contigs. Website databases of GenBank (nr, EST_human, EST_mouse and EST_other databases) Genbank blast [46], European Bioinformatics Institute [47] and Swiss-Prot [48] were also mined by tblasn or blastp using the same query sequence as above [18].

### BAC library screening and BAC DNA sequencing

*S. mansoni *BAC clones containing *SmTRα *and *SmTRβ *were identified by screening *S. mansoni *SmBAC1 or CHOR-1 BAC library with methods previously described [23]. For BAC DNA sequencing, the BAC clone was grown in 100 ml LB medium (12.5 μg/ml choramphenicol) and the BAC DNA was purified using Plasmid Midi kit (Qiagen) and sequenced on an ABI-377 automatic sequencing machine.

### Phylogenetic tree construction

Phylogenetic trees were constructed from deduced sequences of the DBD and the sequence of DBD plus LBD, respectively. Sequences were aligned with ClustalW [49]. Phylogenetic analysis of the data set was carried out using Maximum Likelihood method under Jones-Taylor-Thornton (JTT) substitution model [50] with a gamma distribution of rates between sites (eight categories, parameter alpha, estimated by the program) using PHYML (v2.4.4)) [51] with support values obtained by bootstrapping a 100 replicates. The same data set was also tested by Bayesian inference using MrBAYES v3.1.1 with a JTT amino acid replacement model + gamma rates [52]. The trees were started randomly; four simultaneous Markov chains were run for 5 million generations for the DBD data set and 3 million generations for the DBD+LBD data set, respectively. The trees were sampled every 100 generations, the Bayesian posterior probabilities (PPs) were calculated using a Markov chain Monte Carlo (MCMC) sampling approach implemented in MrBAYES v3.1.1, with a burn-in value setting at 12,500 for DBD data set and 7,500 generations for the DBD+LBD data set, respectively.

### GST Pull-down

cDNAs encoding part of the hinge region plus the LBD domain of SmTRα (Val739-Glu1115) and SmTRβ (Pro487-Cys847) were inserted into pGEX-4T-1 vectors to form pGEX-SmTRα(LBD) and pGEX-SmTRβ(LBD). cDNA encoding SmRXR1 was previously inserted into pCITE-4a vector to form pCITE-SmRXR1 [19]. *E. coli *AD 494 (DE3) pLys S competent cells (Novagen) were transformed with pGEX-SmTRα(LBD) or pGEX-SmTRβ(LBD) and the GST fusion proteins were purified by passage over a glutathione-Sepharose column according to standard protocols. pCITE-SmRXR1 was transcribed and translated using the Single Tube Protein System (Novagen) following the manufacture's protocol to produce ^35^S labeled protein. For pull-down experiments, a 50 μl reaction that contained 2 μl of the *in vitro *translation reaction, SmTRα(LBD) or SmTRβ(LBD) GST fusion protein, or GST protein (negative control) affixed to glutathione-Sepharose beads (about 2 μg) and binding buffer (50 mM Tris-HCl, pH 7.5, 100 mM NaCl, 10% glycerol, 0.15% Nonidet P40) was used [19]. The reaction was incubated overnight at 4°C and then washed three times with binding buffer. The bound proteins were analyzed by 10% SDS-PAGE and autoradiography.

### Electrphoretic mobility shift assay (EMSAs)

cDNA encoding SmTRα, SmTRβ and SmTRα(Gln182-Ala288) (containing 20 aa at the 5' end of the DBD, DBD and 40 aa at the 3' end of the DBD) was inserted into pCITE-4a vector to form pCITE-SmTRα, pCITE-SmTRβ and pCITE-SmTRα(Gln182-Ala288). The protein was produced *in vitro *using TNT quick coupled transcription/translation system (Promega). The following complementary single-stranded oligonucleotides containing consensus half-sites AGGTCA were synthesized:

half-site: 5'-GTACCGTAAGGTCACTCGCGT-3',

DR0: 5'-CCGTAAGGTCAAGGTCACTCG-3',

DR1: 5'-CCGTAAGGTCACAGGTCACTCG-3',

DR2: 5'-CCGTAAGGTCACAAGGTCACTCG-3',

DR3: 5'-CCGTAAGGTCACAGAGGTCACTCG-3',

DR4: 5'-CCGTAAGGTCACAGGAGGTCACTCG-3',

DR5: 5'-CCGTAAGGTCACCAGGAGGTCACTCG-3'.

Pal0: 5'-CGCAAGGTCATGACCTCG-3' [19]. One strand of each oligonucleotide was annealed after incubation at 100°C for 3 minutes to its complementary oligonucleotide and then labeled with T4 polynucleotide kinase and γ-^32^P adenosine triphosphate. The binding reactions were incubated on ice for 40 minutes in 15 μl reaction mixture containing 40,000 cpm of probe, 3 μl *in vitro *translation reaction, 3 μl 5 × buffer (20% glycerol, 5 mM MgCl_2_, 2.5 mM EDTA, 2.5 mM DTT, 250 mM NaCl, 50 mM Tris-HCl (pH 7.5) and 0.25 mg/ml poly(dI-dC)poly (dI-dC)). The reaction was separated on 6% (v/v) native polyacrylamide gel containing 2.5% glycerol in 1 × TBE buffer at 4°C [19]. Gels were dried, exposed to x-ray film and autoradiographed.

## Abbreviations

BAC- Bacterial artificial chromosome;

DBD- DNA-binding domain;

JP- Junction position; 

LBD- Ligand binding domain; 

NR- Nuclear receptor; 

NTSS- N-terminal signature sequence; 

TR- Thyroid hormone receptor; 

WGS- Whole genomic sequence.

## Authors' contributions

WW performed the experiments and analysis. He contributed to the writing and is the primary author of the manuscript. EGN was responsible for the design and analysis of the experiments. He contributed to the writing of the manuscript. PTL was responsible for the overall design, analysis and interpretation of the results. He contributed to the writing and preparation of the manuscript. All authors read and approved the final manuscript.

## Supplementary Material

Additional file 1 List of genomic sequences encoding invertebrate TR homologues. Names of species and accession numbers of their genomic sequences analyzed in this study Click here for file

Additional file 2 List of GenBank accession numbers of cDNAs analyzed in this study. Names of species and accession numbers of their cDNAs analyzed in this study Click here for file
