# Supplementary material for: Thyroid hormone receptor orthologues from invertebrate species with emphasis on Schistosoma mansoni
Source: BMC Evol Biol. 2007 Aug 29;7:150. doi: 10.1186/1471-2148-7-150 (PMC2045677; doi:10.1186/1471-2148-7-150)
Supplement: Additional file 1 — List of genomic sequences encoding invertebrate TR homologues. Names of species and accession numbers of their genomic sequences analyzed in this study [file 1471-2148-7-150-S1.doc]

### Additional file 1 – lists of genomic sequences encoding invertebrate TR homologues

1. ftp://ftp.ncbi.nlm.nih.gov/pub/TraceDB/

**DpTR (*Daphnia pulex* TR):**

gnl|ti|809670116: AZSN16580.g1

gnl|ti|809759225: AZSN47306.b1

gnl|ti|811561298: AZSH109861.y1

gnl|ti|811756049: AZSH179591.y1

gnl|ti|812101916: AZSH219724.y1

gnl|ti|812306342: AZSH243913.y1

gnl|ti|812366577: AZSH254703.x1

gnl|ti|814420664: AZSH381993.x1

gnl|ti|897263268: AZSH434387.y1

gnl|ti|882228974: AZWZ608112.b1

gnl|ti|815047965: AZWZ269420.g1

gnl|ti|814817461: AZWZ156514.b1

gnl|ti|814791074: AZWZ139343.g1

gnl|ti|814471490: AZSH386473.x1

gnl|ti|814578974: AZWZ35147.b1

gnl|ti|901533860: AZSH562669.g1

**LgTR: (*Lottia gigantean* TR):**

gnl|ti|829036218: AZYG584427.g1

gnl|ti|829682765: AZYH130255.g1

gnl|ti|829397238: AZZI216995.y1

gnl|ti|828757380: AZYG506336.g1

gnl|ti|826853865: AZYG46257.g1

gnl|ti|829167968: AZZI147865.x1

gnl|ti|829246726: AZZI178198.x1

gnl|ti|829383482: AZZI212420.y1

gnl|ti|829003273: AZYG568186.b1

gnl|ti|827999195: AZYG275032.g2

**SeTRa (*Schmidtea mediterranea* TRa):**

SAAA-alu07h02.b1.seq

SAAA-aue12f08.g1.seq

SAAA-aqp93e01.g1.seq

**SeTRb (*Schmidtea mediterranea* TRb):**

uin96f11.g1.seq

SAAA-alh87g01.b1.seq

SAAA-aik26f09.g1.seq

ufx83b03.b1.seq

udi85g09.b1.seq

1. <http://www.ncbi.nlm.nih.gov/>

**Human TRa (*Homo sapiens* TRa):** AC068669

**Human TRb (*Homo sapiens* TRb):** AC068669

**Sea urchin TR (*Strongylocentrotus purpuratus* TR):** XM_784395

1. [www.jgi.doe.gov](http://www.jgi.doe.gov/)

**Ciona TR (*Ciona intestinalis* TR):** ci0100142683 (Scaffold v 1.0)

1. <http://lifecenter.sgst.cn/sj.do>

**SjTRa *(Schistosoma japonium* TRa):** Contig_0016212

**SjTRb (*Schistosoma japonium* TRb):** Contig_0030503
