# Supplementary material for: Thyroid hormone receptor orthologues from invertebrate species with emphasis on Schistosoma mansoni
Source: BMC Evol Biol. 2007 Aug 29;7:150. doi: 10.1186/1471-2148-7-150 (PMC2045677; doi:10.1186/1471-2148-7-150)
Supplement: Additional file 2 — List of GenBank accession numbers of cDNAs analyzed in this study. Names of species and accession numbers of their cDNAs analyzed in this study [file 1471-2148-7-150-S2.doc]

### Additional file 2 – lists of GenBank accession number of cDNA analyzed in this study

Alligator TRa (*Alligator mississippiensis* TRa): DQ386683

Alligator TRb (*Alligator mississippiensis* TRb): DQ386685

Bull frog TRa (*Rana catesbeiana* TRa): L06064

Bull frog TRb (*Rana catesbeiana* TRb): L27344

Chicken TRa (*Gallus gallus* TRa): NM_205313

Chicken TRb (*Gallus gallus* TRb): NM_205447

Ciona TR (*Ciona intestinalis* NR1): M_001032486

Clawed frog TRa (Xenopus laevis TRb): M35343

Clawed frog TRb (Xenopus laevis TRb): M35360

DuckTRa (*Cairina moschata* TRa): Z50080

DuckTRb (*Cairina moschata* TRb): Z49151

Eel TRa1 (*Conger myriaster* TRa1): AB183396

Eel TRa2 (*Conger myriaster* TRa2): AB183397

Eel TRb1 (*Conger myriaster* TRb1): AB183394

Eel TRb2 (*Conger myriaster* TRb2): AB183395

Halibut TRa (*Paralichthys olivaceus* TRa): D16462

Halibut TRb (*Paralichthys olivaceus* TRb): D45245

Human TRa (*Homo sapiens* TRa): AB209346

Human TRb (*Homo sapiens*): M26747

Lamprey TR1 (*Petromyzon marinus* TR1): DQ320317

Lamprey TR2 (*Petromyzon marinus* TR2 ): DQ320318

Lizard TRa (*Eublepharis macularius* TRa): AB204861

Lizard TRb (*Eublepharis macularius* TRb)AB204862

Medaka TRa (*Oryzias latipes* TRa): AB114860

Medaka TRb (*Oryzias latipes* TRb): AB114861

Rat TRa (*Rattus norvegicus* TRa): M18028

Rat TRb (*Rattus norvegicus* TRb): NM_012672

Human RARa (*Homo sapiens* RARa): X06614

Human RARb (*Homo sapiens* RARb): X07282

Human RARg (*Homo sapiens* RARg): M24857

Human RORa (*Homo sapiens* RORa): U04897

Human RORb (*Homo sapiens* RORb): Y08639

Human RORg (*Homo sapiens* RORg): U16997
